# Taking a new look at how flies learn

**DOI:** 10.7554/eLife.03978

**Published:** 2014-08-19

**Authors:** Benjamin Kottler, Bruno van Swinderen

**Affiliations:** 1 **Benjamin Kottler** is in the Queensland Brain Institute, The University of Queensland, Brisbane, Australiab.kottler@uq.edu.au; 2 **Bruno van Swinderen** is in the Queensland Brain Institute, The University of Queensland, Brisbane, Australiab.vanswinderen@uq.edu.au

**Keywords:** associative memory, dopamine neurons, visual learning, *D. melanogaster*

## Abstract

Learning based on what a fruit fly sees or what it smells might not involve distinct parts of the brain, as was previously thought.

**Related research article** Vogt K, Schnaitmann C, Dylla KV, Knapek S, Aso Y, Rubin GM, Tanimoto H. 2014. Shared mushroom body circuits underlie visual and olfactory memories in *Drosophila*. *eLife* **3**:e02395. doi: 10.7554/eLife.02395

**Image** Specialised equipment was used to train fruit flies to associate different colours with either a punishment (an electric shock) or a reward (sugar)

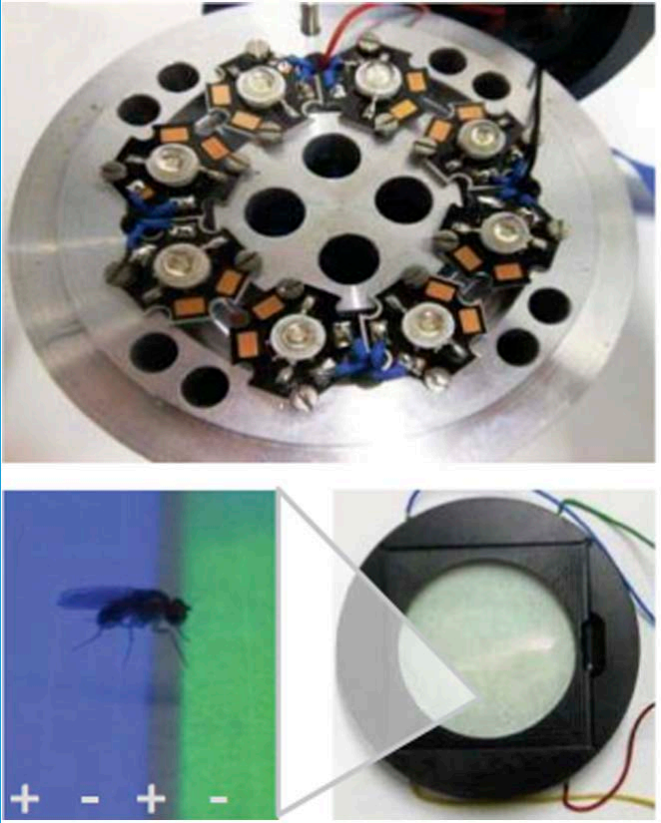

What we see often depends on what we looked for. The same is true for what we understand about the regions of the brain that are involved in learning and memory. For example, fear conditioning—when an animal learns to avoid harmful events based on signals that might predict the event—has been associated with a region of the brain called the amygdala in rats, while experiments in which rats have to learn the route through a maze suggest that the hippocampus is involved (reviewed in Squire, 2004). However, it can be difficult to compare the results of experiments on various aspects of memory and learning because the methods used to obtain the results can be very different.

The same is true in simpler animals such as the fruit fly, *Drosophila melanogaster*. Learning to associate an odour with harmful or rewarding stimuli has been associated with a pair of structures in the fly's brain called the mushroom bodies, whereas visual learning has been mapped to a different brain region called the central complex (reviewed in Kahsai and Zars, 2011). Again, it is difficult to combine and compare the results of experiments on visual learning and olfactory (odour-based) learning because the two experimental approaches are different.

Now, in *eLife*, Hiromu Tanimoto and co-workers at the Max Planck Institute for Neurobiology, the Janelia Farm Research Campus and Tohoku University—including Katrin Vogt and Christopher Schnaitmann as joint first authors—have used a newly developed visual learning assay for *Drosophila* that is similar to the assay widely used in olfactory experiments. This new assay suggests that learning in the flies based on either visual and olfactory signals use overlapping neuronal pathways (Vogt et al., 2014).

With hindsight, it seems intuitive that the brain would have a common mechanism that is able to handle different kinds of memory, whether they are pleasant or unpleasant, or whether they originate from different senses. Researchers working on olfactory learning in flies traditionally use sugar as a reward, and electric shocks as a punishment, to induce positive and negative associations with various scents. The flies are then tested to see if they have learnt these associations by giving them a choice of two odours that they can walk towards after training (Figure 1A). In contrast, experiments on visual learning have traditionally involved flying animals (which are tethered) and used heat as a punishment (Figure 1B). Furthermore, in this flying paradigm the flies themselves can control if they get punished or not by flying in one direction or the other (termed ‘operant’ conditioning). Again, it is not surprising that such completely different tests have associated distinct brain regions with learning behaviour.

**Figure 1.**
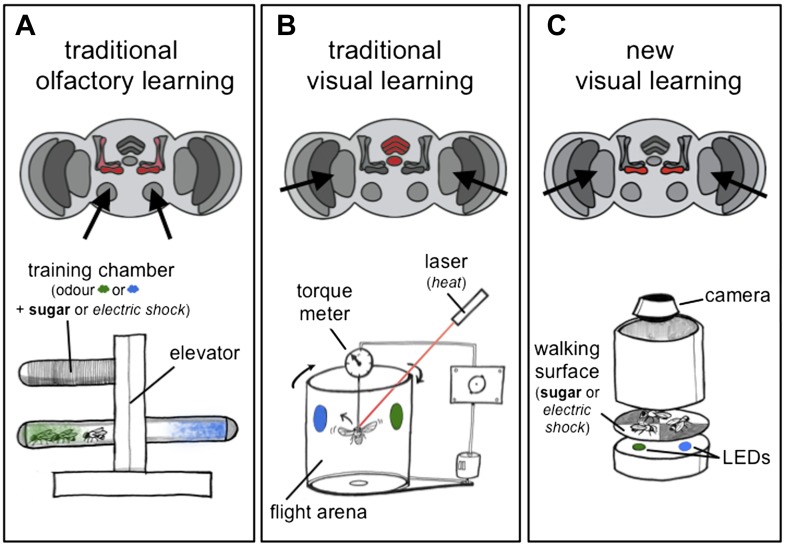
Olfactory and visual learning assays in *Drosophila*. (**A**) Top: odours are first processed via the antennal lobes (arrows), and the mushroom bodies (red) are required for the formation of olfactory memories. Bottom: the ‘T-shaped’ apparatus has been used for most experiments on olfactory learning in flies: odours are either associated with electric shocks (as a punishment, in *italics*) or sugar (as a reward, in **bold**) in the top ‘training chamber’. Groups of flies are then lowered to a choice-point and can decide to walk into one of two chambers that contain different odours. (**B**) Top: visual stimuli are first processed in the optic lobes (arrows), and the central complex (red) is required for the formation of visual memories. Bottom: the tethered flight arena has been used for most experiments on visual learning in flies. A single fly is held in place, but its direction of flight is measured using a torque meter. This in turn controls the position of visual objects (blue and green regions) in the flight arena as the fly moves left or right, towards or away from the objects. Learning is achieved by punishing the fly (by laser heating) when it turns towards one of the objects (the blue region in this case). (**C**) Top: Vogt, Schnaitmann et al. have revealed that the gamma lobes of the mushroom bodies (red) are also associated with visual learning in walking flies. Bottom: a new visual learning assay that can reward or punish groups of flies walking on a surface over coloured lights/LEDs. Flies are not drawn to scale.

Several years ago Tanimoto and co-workers built a visual learning assay that more closely resembled the assays used in olfactory experiments: in this assay flies displayed their learned choices by walking to coloured regions on a platform (Schnaitmann et al., 2010). Vogt, Schnaitmann et al. have now made this assay even more similar to olfactory learning tests, by also using electric shocks as the punishment and sugar as the reward (Figure 1C). However, to be able to punish or reward the flies on the same surface that presents the visual cues has required some innovative engineering.

First, Vogt, Schnaitmann et al. showed that flies could be trained to avoid or prefer a visual cue (coloured lights). Then, they probed the neurons involved in a classical olfactory learning pathway to investigate whether any of these neurons could also process visual memories. A substantial overlap was found: the dopamine neurons that signal punishment or reward for odour learning (Waddell, 2010) and the dopamine receptor that communicates these signals to the mushroom bodies are also required for visual learning. Moreover, the ‘gamma lobes’ of the mushroom body appear to be involved in both short-term olfactory memories and short-term visual memories, while other neurons in the mushroom body show distinct effects.

So, how do we reconcile these new results with decades worth of *Drosophila* research pointing to the mushroom bodies as centres for olfactory memories? First, the work highlights the importance of behavioural context. Earlier work using flying insects (Figure 1B) suggested that the mushroom bodies are not needed for simple visual learning, but are instead required for learning that the same signal or stimulus can have the same meaning in different contexts (e.g., learning that an object can be presented on a blue background or a green background and still be the same object; Wolf et al., 1998; Liu et al., 1999). By showing that mushroom body circuits are indeed involved in simple visual learning, Vogt Schnaitmann et al. raise the possibility that walking flies and flying flies might use different memory pathways.

Another way that tethered flight and walking paradigms are different for visual learning is the fact that visual stimuli are much better controlled in the former, because the fly always sees the stimuli in the same way (Brembs and Wiener, 2006). As such, walking assays for measuring visual or olfactory learning might implicate the mushroom body precisely because these neurons may be needed by the fly to generalise the context, which is always changing when the flies are free to walk around. Alternatively both the mushroom bodies and the central complex might be required for visual learning in this new assay, which remains a possibility because Vogt, Schnaitmann et al. did not probe central complex neurons.

In mice and rats, the amygdala, medial prefrontal cortex and hippocampus interact to guide behavioural choices (Maren et al., 2013). It is therefore likely that a network of different structures in the fly brain also interact to guide learned behaviours.

Another possible explanation for the overlap found between visual and olfactory learning is that certain circuits in the mushroom body, such as the dopamine-to-gamma-lobe pathway highlighted by Vogt, Schnaitmann et al., are involved in ‘selective attention’ (van Swinderen et al., 2009). This process describes when an animal concentrates on one aspect of its environment, and the same circuits might be involved regardless of which sense is providing the information (e.g. sight or smell). The mushroom bodies may still respond only to olfactory cues or the central complex to visual cues, but both probably require a common filtering mechanism to allow the fly to decide what to do. By making the assays that test different aspects of memory and learning more comparable, Vogt, Schnaitmann, Tanimoto and co-workers have now placed *Drosophila* researchers in a better position to understand how the fly brain makes these decisions.
